# An image dataset of fruitfly species (Bactrocera Zonata and Bactrocera Dorsalis) and automated species classification through object detection

**DOI:** 10.1016/j.dib.2022.108366

**Published:** 2022-06-09

**Authors:** Sana Tariq, Ayesha Hakim, Awais Ahmad Siddiqi, Muhammad Owais

**Affiliations:** a Department of Computer Science, Muhammad Nawaz Shareef University of Agriculture (MNSUA), Multan, Pakistan; b School of Software, Tsinghua University, P.R. China

**Keywords:** Fruitfly, Bactrocera Zonata, Bactrocera Dorsalis, Microcontroller, Smart trap, IoT, Artificial intelligence, Object detection

## Abstract

This data article describes the image dataset collection and annotation of the two most common fruitfly species *Bactrocera Zonata* and *Bactrocera Dorsalis*. The dataset is released as a collection of more than 2000 images captured through two sources: images of specially reared fruitfly species in laboratory captured by (48-megapixels) smartphone camera, and images of fruitflies captured by (8-megapixels) Raspberry Pi camera through insect traps installed in fruit orchards. Each image sample is associated with a ground truth label that mentions the fruit fly species. The dataset has been classified and annotated using the object detection method into two fruitfly species with an average 85% accuracy. The results of classification and annotation have been validated by expert entomologists by manually examining test samples in a laboratory setting. This dataset is best suited for developing smart monitoring systems to provide advisory services to farmers through mobile applications that provides real-time information about fruitfly species for effective control and management.

## Specifications Table

**Table:** 

Subject	Bioinformatics
Specific Subject area	Computer Science. Computer vision and Pattern Recognition (One of the techniques of computer vision is object detection. This technique allows to locate and identifies the objects in an image. Object detection can be used to count the objects in an image or video.)
Type of data	Images and Text Files
How data were acquired	Data were captured by using a smartphone camera (48-megapixels Oppo F11) and Raspberry Pi camera (8-megapixels)
Data format	Analysed
Parameters For data collection	The data were collected to develop automated system for identification of two fruit fly species (*Bactocera Zonata and Bactocera Dorsalis)* that attack a fruit or vegetable orchard so that farmers can take precautionary measures against them. These data can also be used to study and analyze the behavior of fruit flies.
Description of data collection	The dataset consists of images and text files. Images were captured in two ways: lab environment by specially rearing fruit flies and in the field by capturing fruit flies through a trap. Images in the lab were captured using smart phone camera 48-megapixel both in daylight and room settings, and images in the field were captured using Raspberry Pi camera 8-megapixel camera. Most of the images contains both species of fruit flies. .JPG format is used for all images and .TXT for text files. The text files contain information about the number of flies, type of species and location of flies in the field.
Data source location	• Institution: Muhammad Nawaz Shareef University of Agriculture, Multan • City/Town/Region: Multan/ Punjab • Country: Pakistan • Latitude and longitude (and GPS coordinates, if possible) for collected samples/data: 30.1475° N, 71.4436°
Data accessibility	Repository name: An Image Dataset of Fruit fly Species Data identification number: doi:10.17632/hgz2n5jxhp.1 Direct URL to data: http://dx.doi.org/10.17632/hgz2n5jxhp.1

## Value of the Data

• This dataset is not only useful for fruitflies species identification, but also to study and analyze the behavior of fruitflies species. By using this dataset, farmers and entomologists can save their time and effort to frequently visit orchards or fields.
• Timely identification of fruitflies using the given dataset can help save 40-50% loss of fruits and vegetables caused by attack of fruitflies.
• This dataset can be used for the detection of fruit fly species in any specific area. Temperature, humidity, and moisture sensors data can be related to the growth rate and population density of fruit flies.
• The data can be used by entomologists and relevant researchers to study the relation between population density of fruitflies with changing climate.

## 1 Data Description

This dataset consists of images and text files. Images were captured using two different quality cameras: Raspberry Pi camera 8 megapixel and smart phone camera 48 megapixel. Most of the images contains both species of fruit flies. JPG format is used for all images. The text files contain information about the number of flies, type of species and location of flies in the field.

**Fig. 1:**
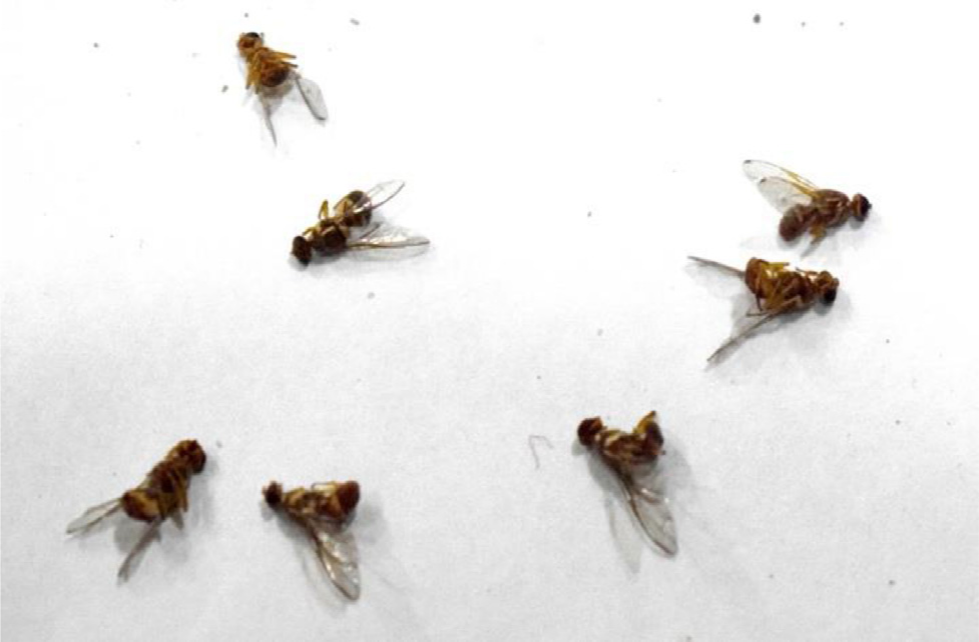
Image file.

**Fig. 2:**
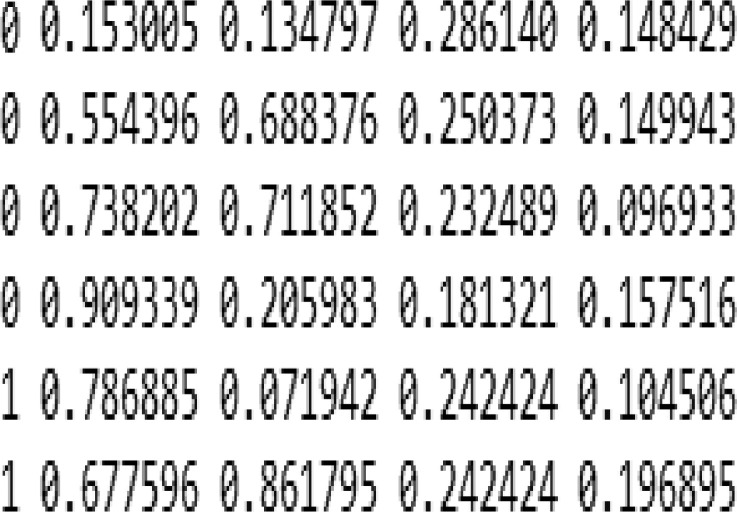
Text file.

Fig. 1 shows fruitflies at different positions in the image. The related text file is shown in Fig. 2 in which 0 represent *Bactocera Dorsalis* and 1 represents *Bactocera Zonata.* Other values in the text file shows the axis position of each fly in the image. This dataset consists of 2000 images and 2000 text file related to images. There is one other file namely class that is also in the text format. This file contains the name of classes or objects that needs to be classified as *‘Zonata’* and ‘*Dorsalis’*

## 2 Experimental Design, Materials, and Methods

The proposed system for automated identification of fruitfly species is based on five phases:

(1) Dataset Generation
(2) Dataset Annotation
(3) Image Pre-processing
(4) Species detection and classification through object detection techniques
(5) Developing Android app for the end-user

### 2.1 Dataset Generation

**Fig. 3:**
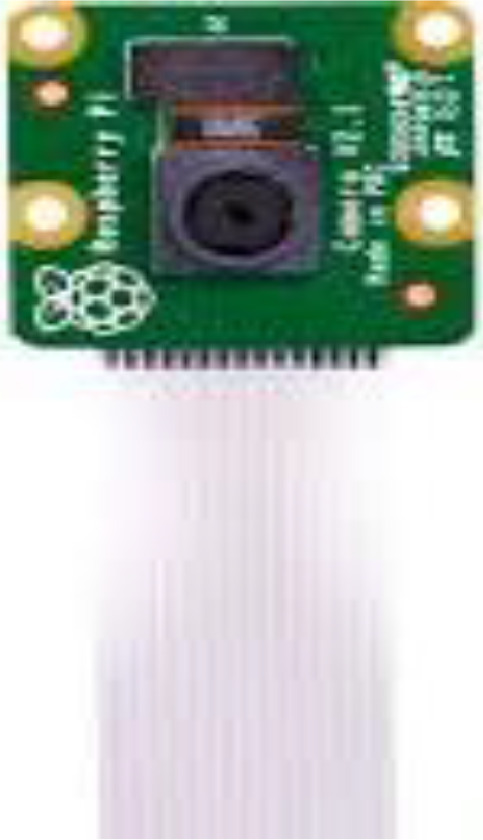
Raspberry Pi Camera (8-megapixel).

Agricultural datasets are not readily available due to lack of technical expertise in this domain. Due to unavailability of reasonable datasets, it is hard to develop automated systems for monitoring and surveillance of insect pests and various crop diseases [1]. Our dataset contains 2000 images of mango fruit fly species namely *Bactocera Zonata and Bactocera Dorsalis*. An object detection technique (YOLO v5) has been used to detect and identify both species [2]. The original images have been enhanced by performing data augmentation techniques. Images are captured in different backgrounds with varying lighting conditions. The dataset was captured manually using a smartphone camera (48 megapixels) and automatically through a smart trap installed in the field using raspberry pi camera (8 megapixels) [3] as shown in Fig. 3.

**Fig. 4:**
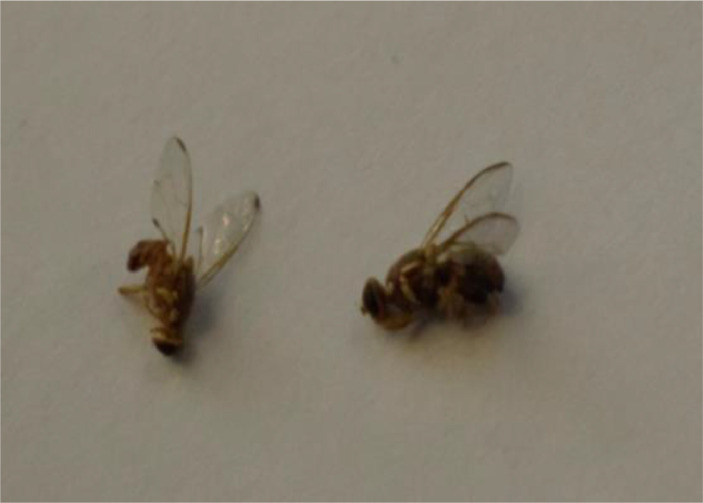
Images captured in the field through Smart Trap.

**Fig. 5:**
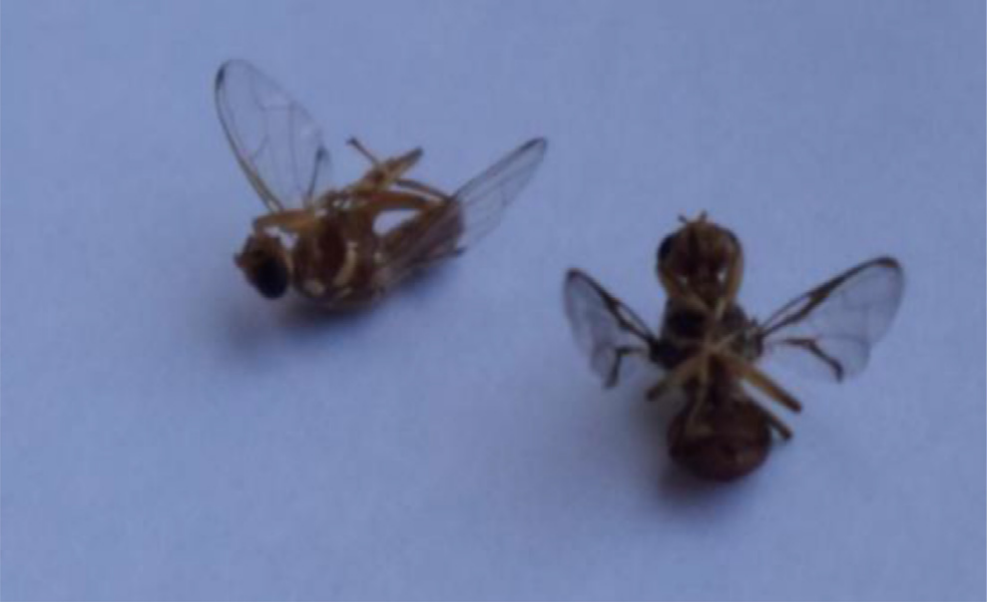
Images captured in laboratory settings.

Data Collection in Insect Rearing Laboratory:

In insect rearing laboratory, fruit flies were reared by providing optimal growth conditions under supervision of entomologists. Images were captured using a smartphone camera (48-megapixels). Fig. 4 contains images of both species of fruit flies *Bactrocera Dorsalis* and *Bactrocera Zonata*, captured in the daylight through smart trap installed in the field. Fig. 5 presents images captured in the laboratory environment.

**Fig. 6:**
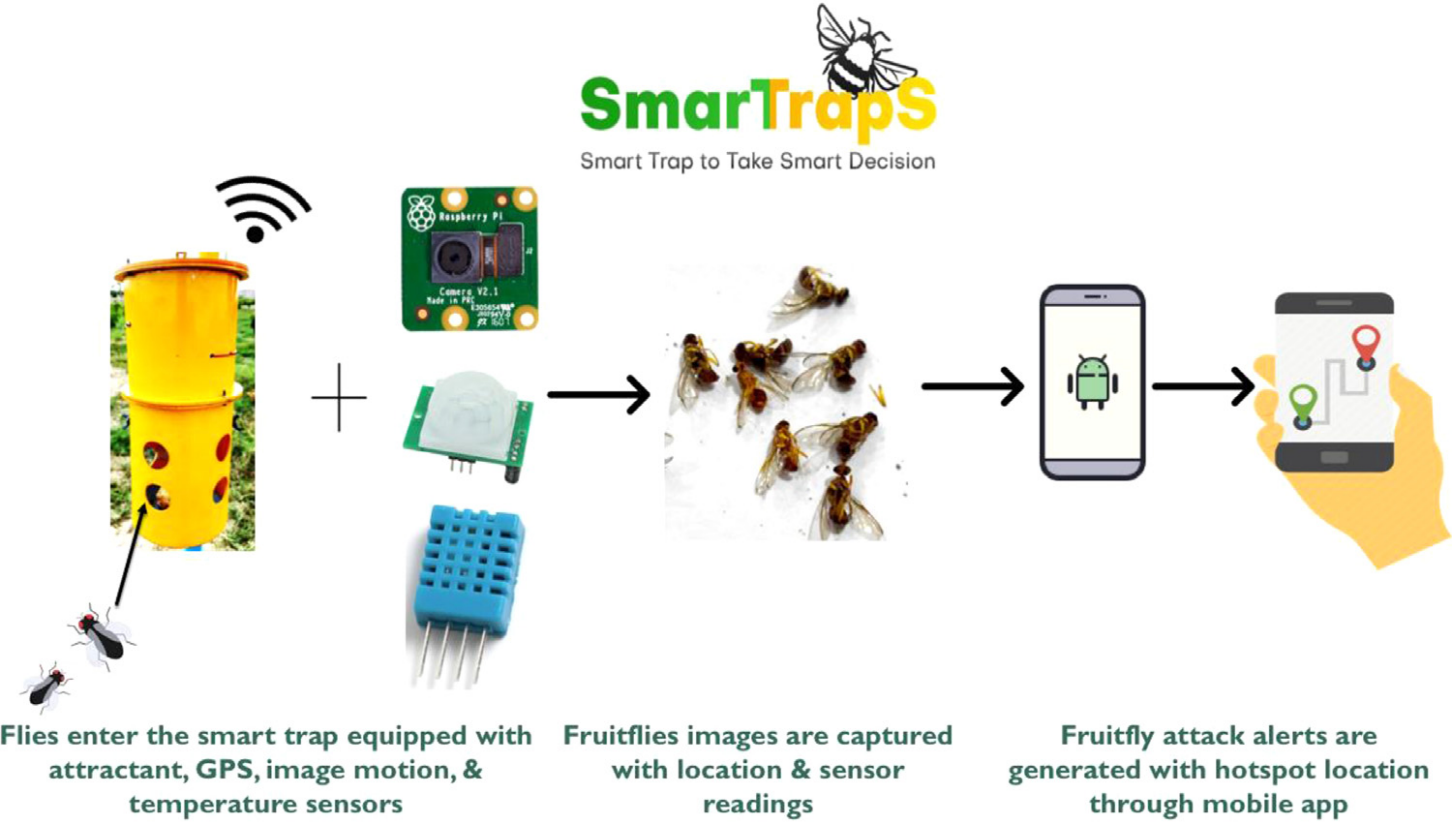
Data Collection using Smart Trap installed in a local fruit orchard.

Data Collection in Fruit Orchards:

A smart trap has been used to collect images of fruit flies in orchards. The trap is equipped with a camera, minicomputer Raspberry Pi [4], temperature sensor [5], humidity sensor, and attractant material to attract fruit flies. The methodology is shown in Fig. 6.

*Methyl Eugenol* was used as an attractant material to attract fruit flies that is proved to be an effective attractant for these species [6]. The camera captures the images of fruit fly from the trap and stores these images on the cloud server. Images that are stored on the server were further used for the validation purpose of automated species detection and identification.

**Fig. 7:**
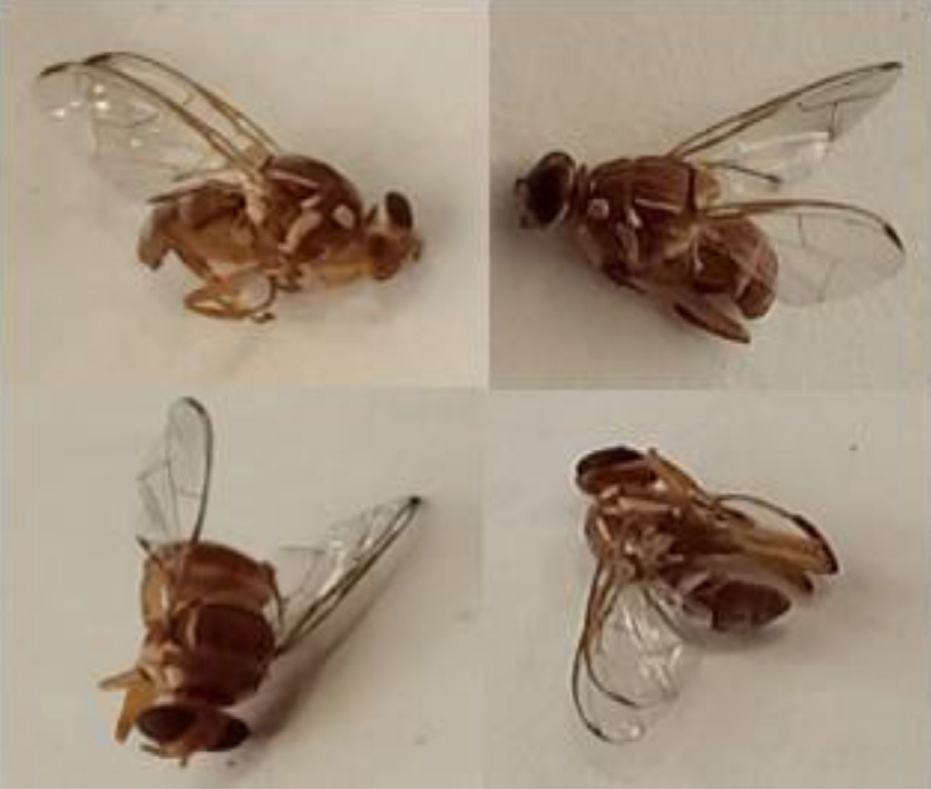
Bactrocera Dorsalis.

**Fig. 8:**
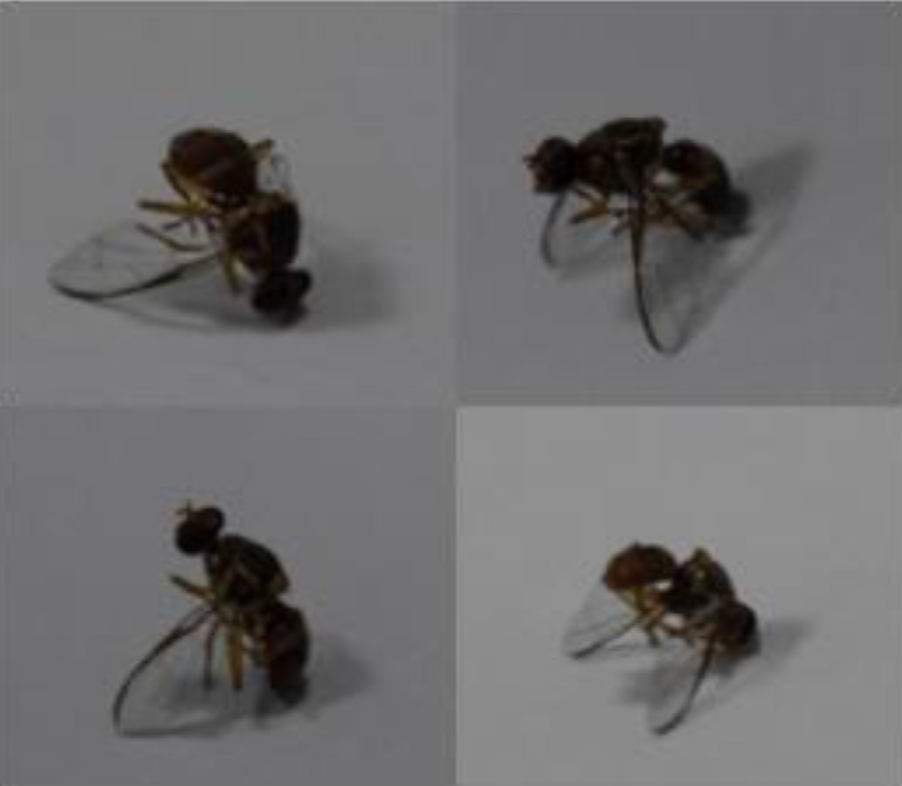
Bactrocera Zonata.

*Bactrocera Dorsalis* is a common pest found in around 65 countries and is known to cause huge damage to fruits and vegetables. It has yellow markings on the thorax and a dark T-shaped marking on the abdomen [7]. *Bactrocera Dorsalis* [8] contains the complete costal band and complete line on the abdomen. This dataset contains images of both *Bactrocera Dorsalis (*Fig. 7*) and Bactrocera Zonata* (Fig. 8) at different angles.

**Fig. 9:**
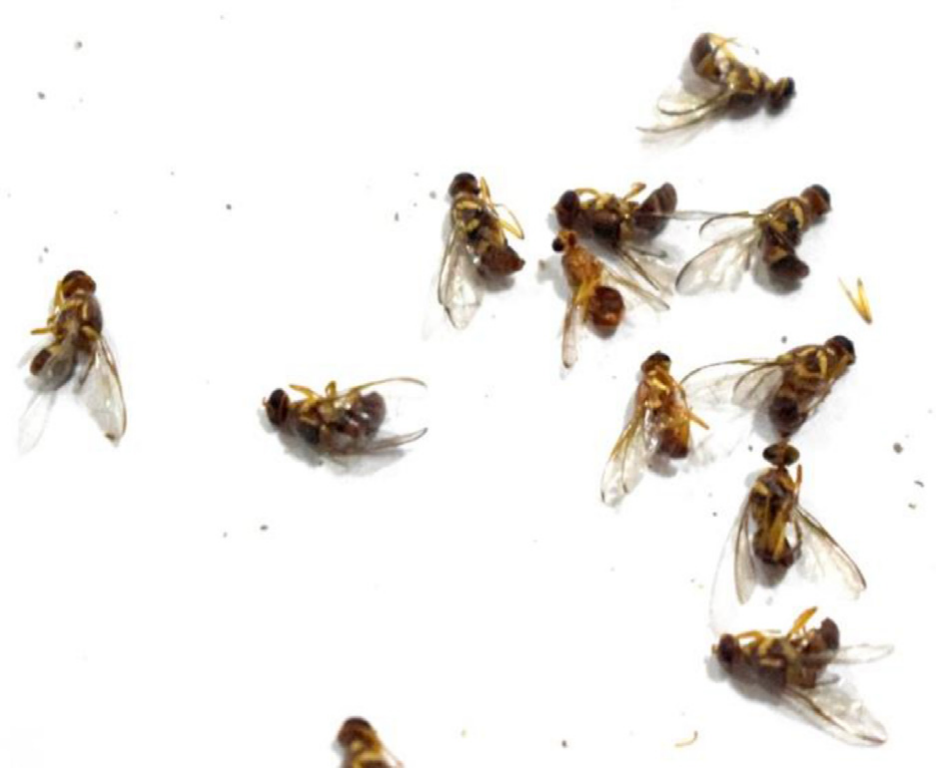
Multiple fruitflies in an image.

*Bactrocera Zonata*[9] is radish brown in color with an apical spot on the end of the wings. It does not contain a costal band on the wing. In the dataset, most of the images contain both species of fruit flies and some images contain only one species either *Bactrocera Dorsalis or Bactrocera Zonata.* Some images contain more than 10 fruit flies as captured from the trap installed in the mango orchards. An example of image containing multiple fruit flies at a time is shown in Fig. 9.

### 2.2 Data Annotation

The dataset was annotated using *labelImg* tool [10] that is free open-source software for labeling images. It supports image labeling in different file formats such as PASCAL, VOC, XML, and YOLO TXT. Two classes were entered in this software naming ‘*Dorsalis’* and ‘*Zonata’*.

**Fig. 10:**
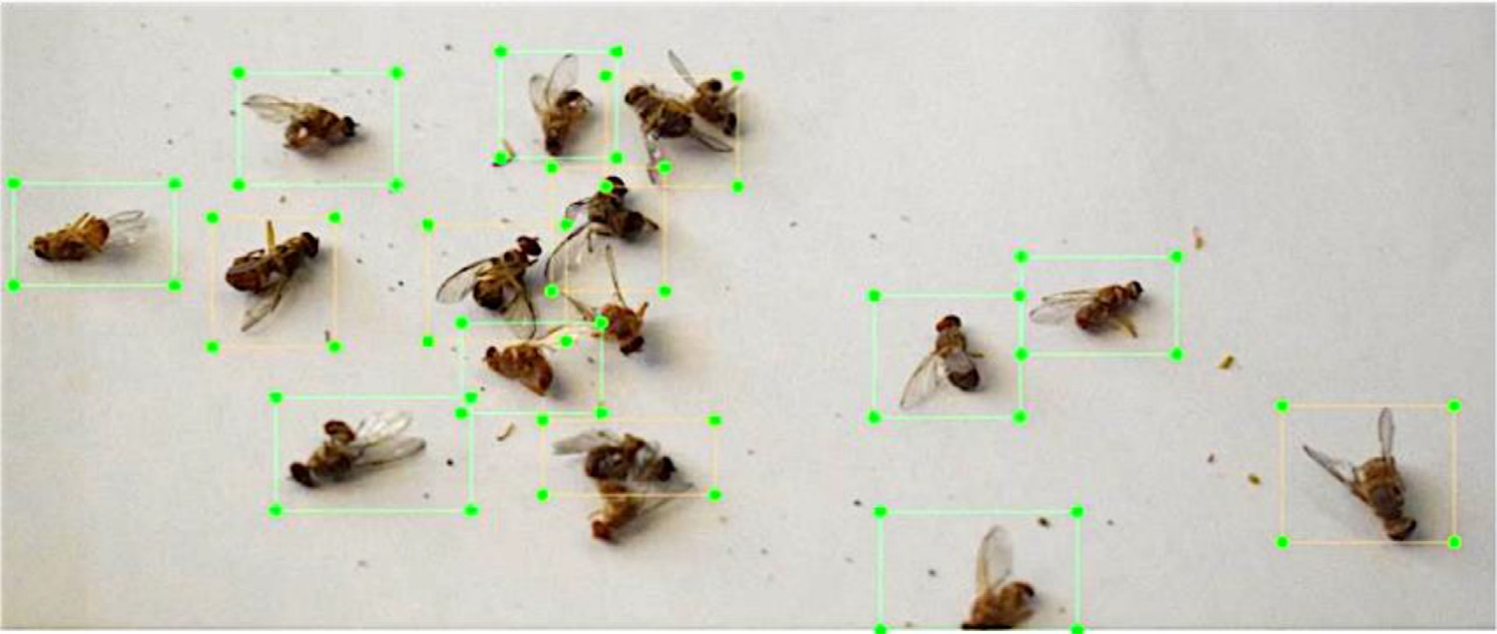
Image annotation.

Fig. 10 presents the data annotation by creating bounding boxes around each fly and label it with respect of the class to which it belongs. This labeling has been done by expert entomologists. The bounding box format of YOLO TXT was used in labeling to proceed with the training the YOLO object detection model.

### 2.3 Image Processing

**Fig. 11:**
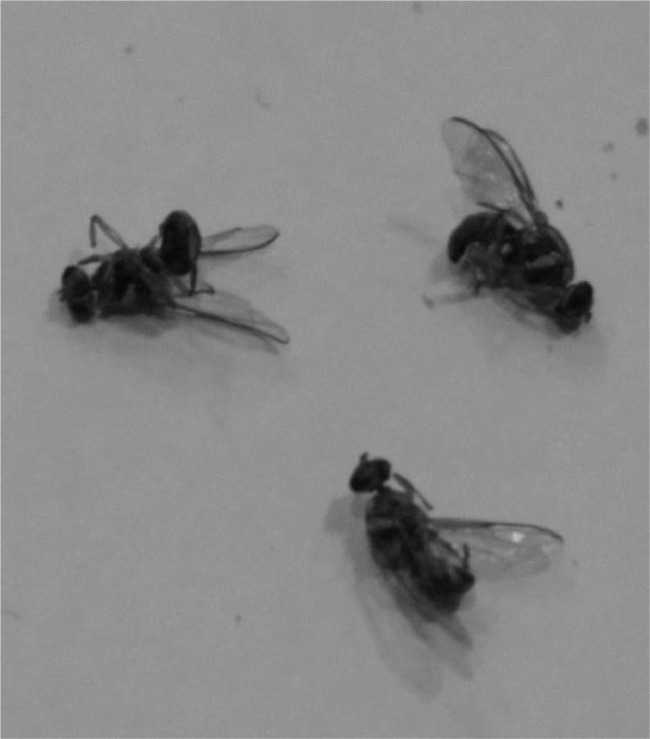
Grayscale image.

**Fig. 12:**
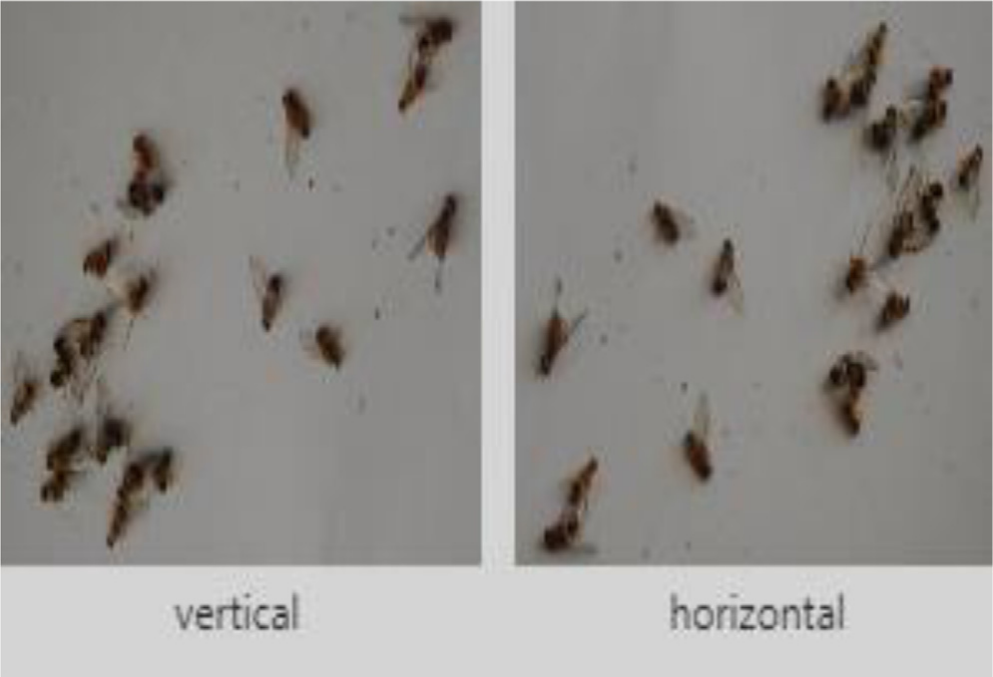
Flipping.

**Fig. 13:**
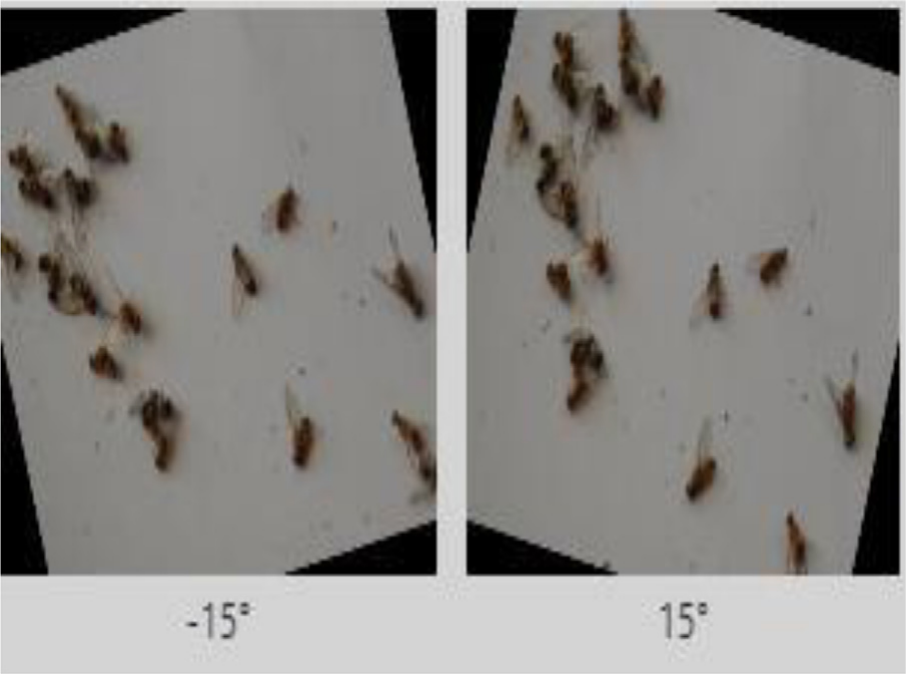
Rotation.

Images were preprocessed using *roboflow* [11] that enables developers to build their computer vision applications with little or no expertise. Images and their labels were uploaded on *roboflow* and preprocessing was performed on these images. We applied preprocessing techniques of resizing and augmentation (flip, rotation, crop, brightness, saturation, noise removal) on the dataset. All images were resized to 416 × 416 and converted into grayscale (as shown in Fig. 11). Flipping has been done horizontally and vertically as shown in Fig. 12. Rotation has been done at an angle of ±15 degrees as shown in Fig. 13.

The dataset contains 2000 images, out of which 70% of images were used for training, 20% for testing, and 10% for validation. The data was exported in YOLOv5 format using *roboflow*.

### 2.4 Species Detection and Classification using Object Detection Technique

Artificial Intelligence (AI) is a branch of computer science that deals with developing machines or robots that mimic human intelligence to perform their tasks. Machine learning is the subfield of artificial intelligence, and the subfield of machine learning is deep learning. Computer vision is the branch of AI where the computer can visualise the world and analyze the visual data to make decisions. One of the main reasons behind the advancement of computer vision is that a large amount of visual data (including images, videos) is being produced by the world, especially after the emergence of smart phones. Object detection is a computer vision technique that helps us to identify and locate the objects in an image. This technique creates bounding boxes around the object of interest to locate the detected object among multiple objects.

YOLO (You Only Look Once) is a family of object detection models that was introduced in 2016 [12]. YOLOv5 is a single-stage object detector that has the following parts namely model backbone, model neck, and model head. Model backbone uses the Cross stage partial network (CSP) to extract the important features from the given input image. Model neck generates the feature pyramids that help to identify the same objects with different sizes and scales and helps a model to perform well on unseen data. In YOLOv5, PANet feature pyramids technique is used at the model neck. The model head part of YOLOv5 is the same as the previous versions. It generates the bounding boxes and class probabilities. In YOLOv5, Leaky Rectified Linear Unit (ReLU) and Sigmoid has been used as activation functions.

### 2.5 Result and Analysis

For training of YOLOv5 object detection model, Google Colab with GPU was used. This model can be trained on CPU, but GPU increases the processing speed of the model. The weights of trained model were stored on the server.

**Fig. 14:**
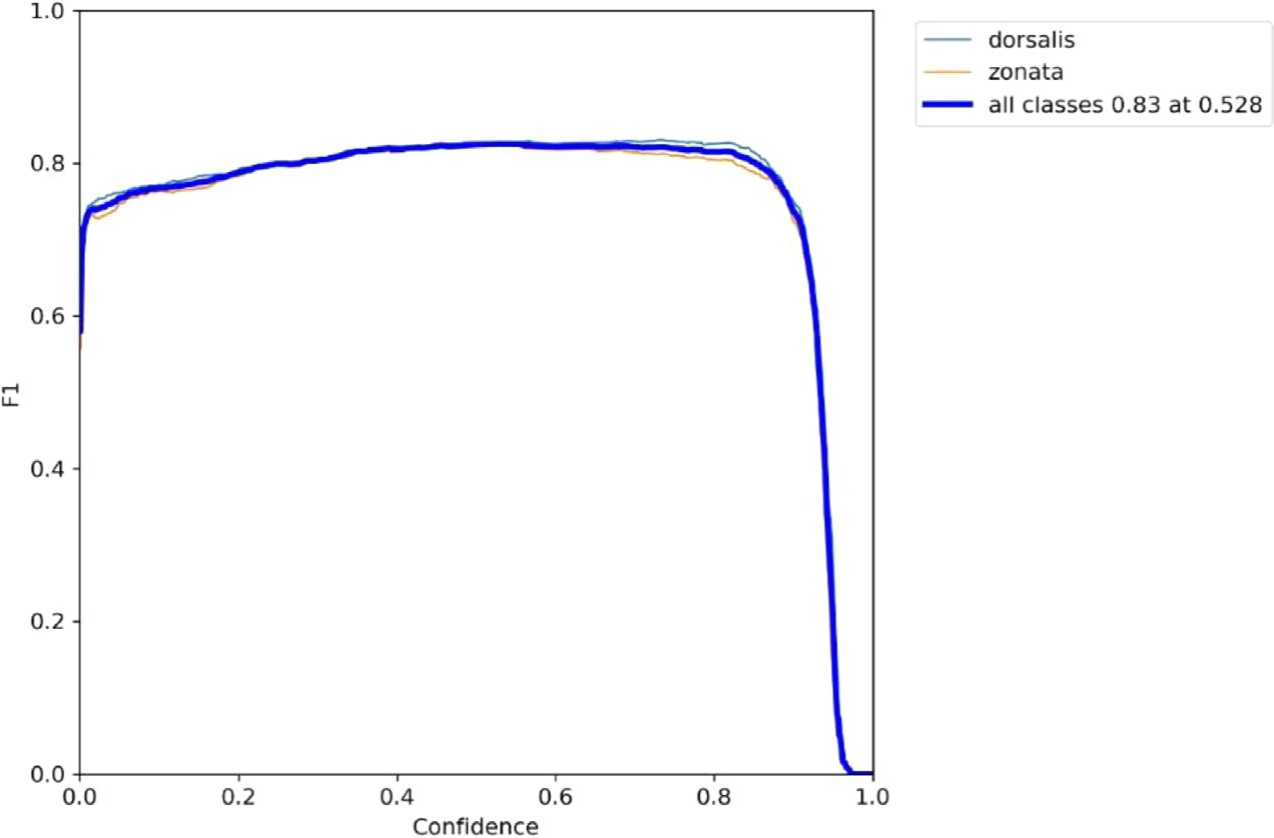
F1-score for detection of Bactrocera Dorsalis (blue line) and Bactrocera Zonata (red line).

Training:

YOLOv5 repository was cloned and computer vision library PyTorch was imported into the Colab file. The dataset is labelled into two classes ‘dorsalis’ and ‘zonata’ that refers to *Bactrocera Zonata* and *Bactrocera Dorsalis* respectively. We trained our dataset with 2000 epochs that achieved maximum performance. After training, we evaluated our training process by analyzing matrics such as F1 score and confusion matrix. F1 score (also known as F score or F measure) is a measure of model accuracy on the given dataset and is the weighted average of precision and recall. It is measured by the following equation, where precision is the division of true positive results and the number of all positive results, and recall is the ratio of true positive results to all observations in the actual class. In Fig. 14, blue line represents *Bactrocera Dorsalis* and red line represents *Bactrocera Zonata* achieving F1-score of 0.83.

$$F1\;\text{score}=\;\frac{2\;\times \left(\text{Precision}\;\times \text{Recall}\right)}{\text{Precision}+\text{Recall}}$$

The performance of a classification model can be analysed by a table or matrix that is known as confusion matrix. Following terminologies are used in the confusion matrix:

True Positive: When the model predicted positive and it is true.
True Negative: When the model predicted negative and it is true.
False Positive: When the model predicted positive and it is false.
False Negative: When the model predicted negative and it is false.

**Fig. 15:**
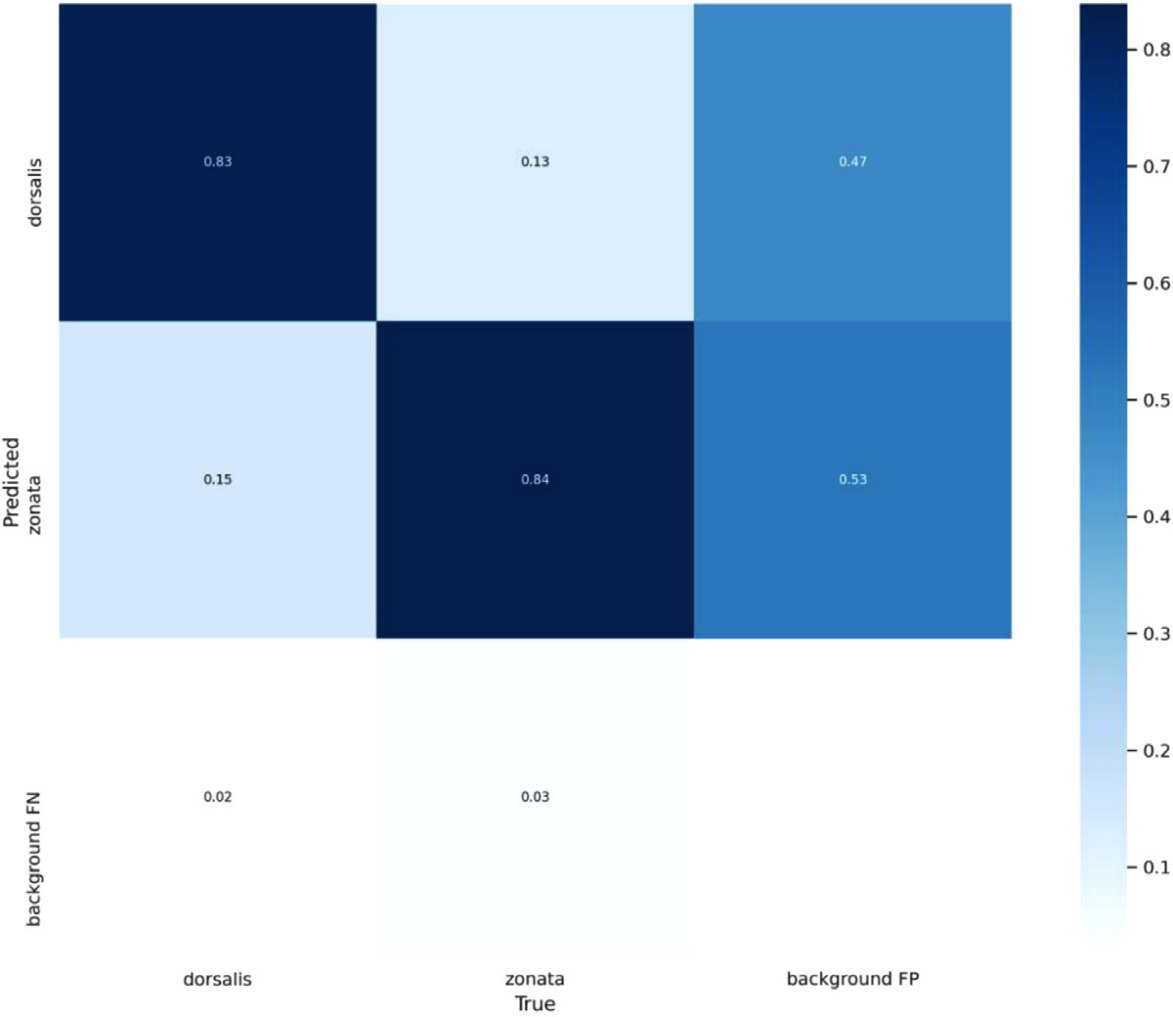
Confusion Matrix for the proposed object detection model.

Fig. 15 presents confusion matrix for our object detection model. This confusion matrix shows that YOLOv5 predicted *Bactrocera Dorsalis* with an average accuracy of 83% and predicted *Bactrocera Zonata* with an average accuracy of 84%.

## Testing

**Fig. 16:**
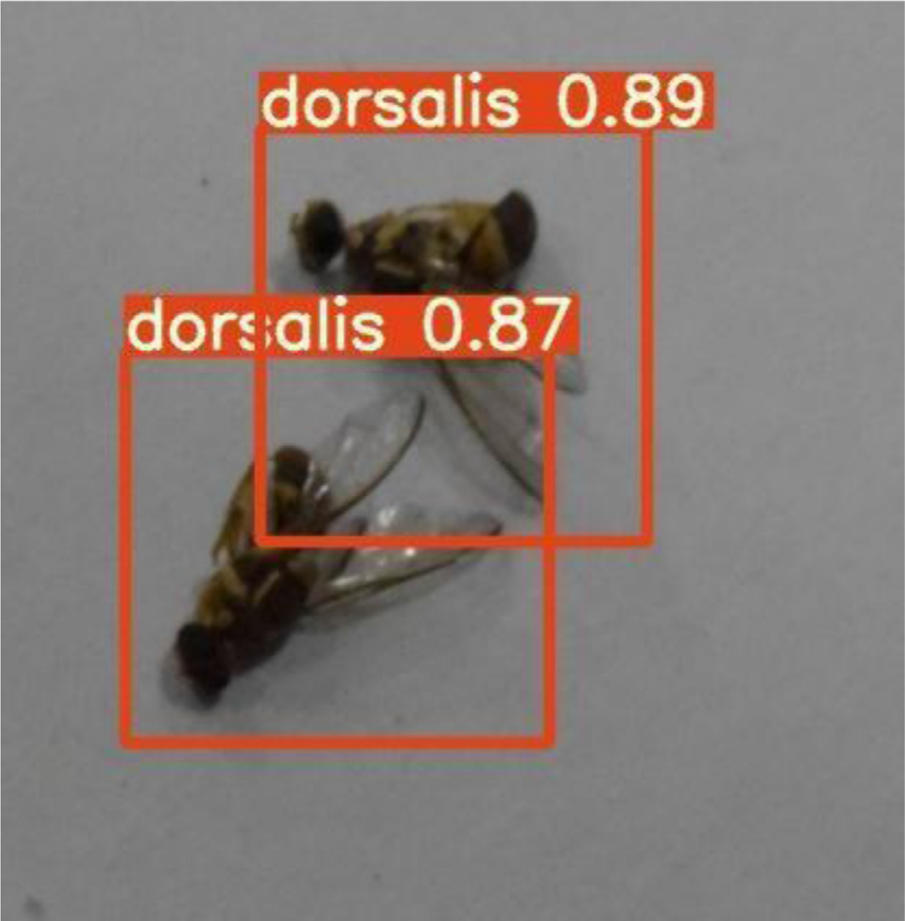
Testing result with the same species.

**Fig. 17:**
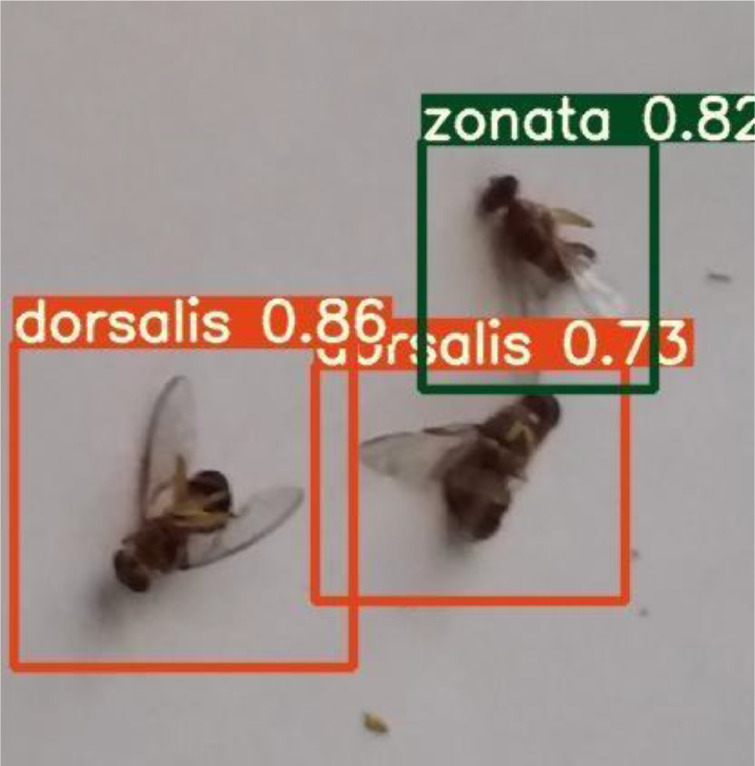
Testing result with mixed species.

YOLOv5 is fast and accurate as compared to its previous versions. It only takes an average 0.009sec to classify a test image from the testing dataset. Figs. 16 and 17 shows some of the results of testing using YOLOv5.

Fig. 16 shows that YOLOv5 detects the *dorsalis* species and draws the bounding boxes with confidence level and a label. Fig. 17 shows that our system detects both species of fruit flies.

**Fig. 18:**
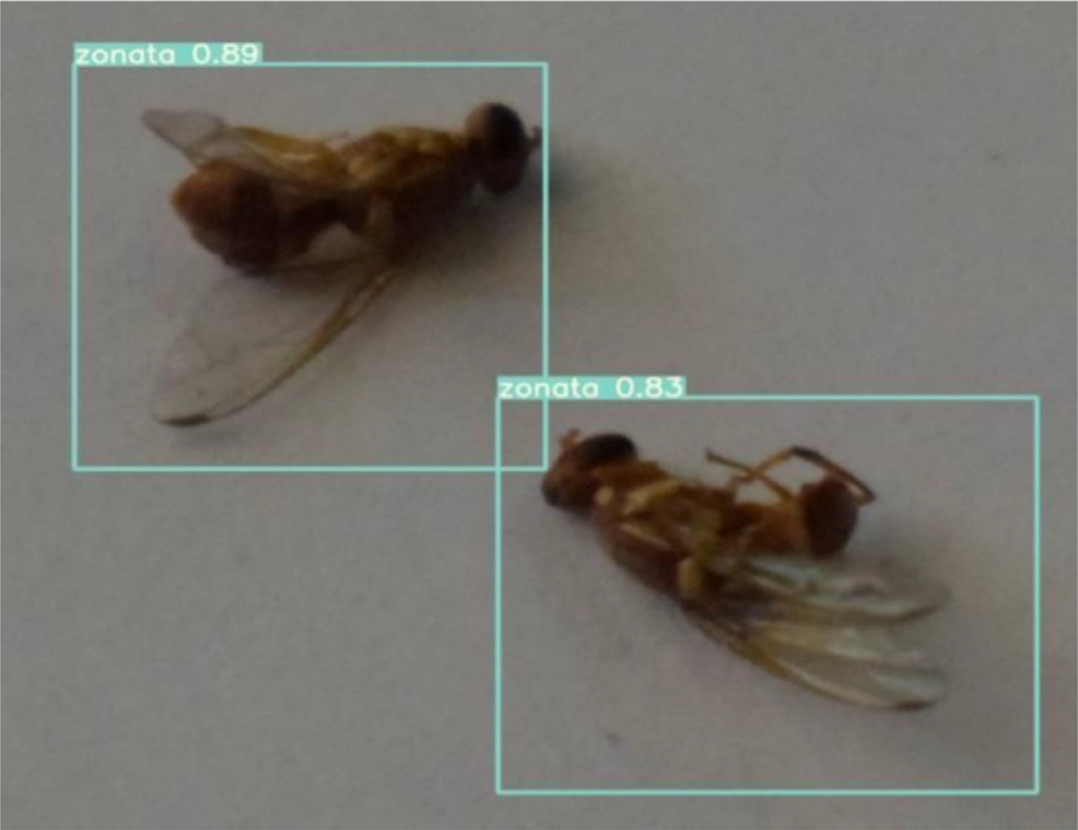
Validation of same species image.

**Fig. 19:**
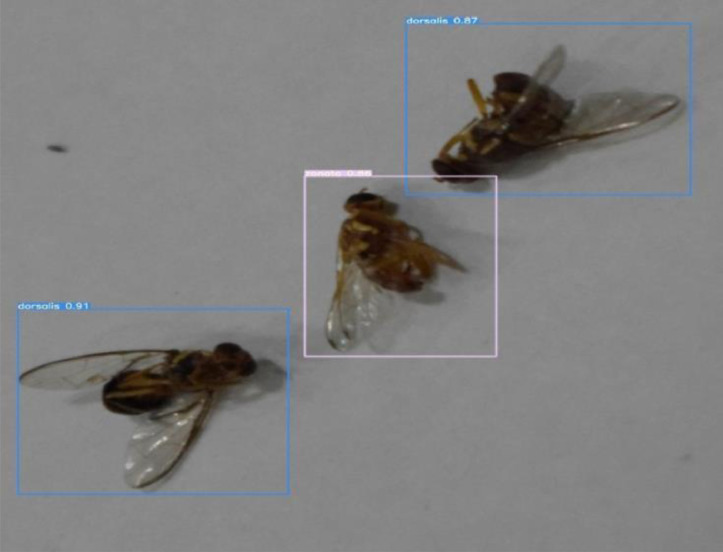
Validation of mixed species image.

Validation:

After training and testing, the weights obtained from YOLOv5 for our custom dataset were transferred to the server. These weights are used for validation and inference. The images after species detection and recognition can be visualized using mobile application. Figs. 18 and 19 shows the result of unseen images that are never seen by our trained model.

**Fig. 20:**
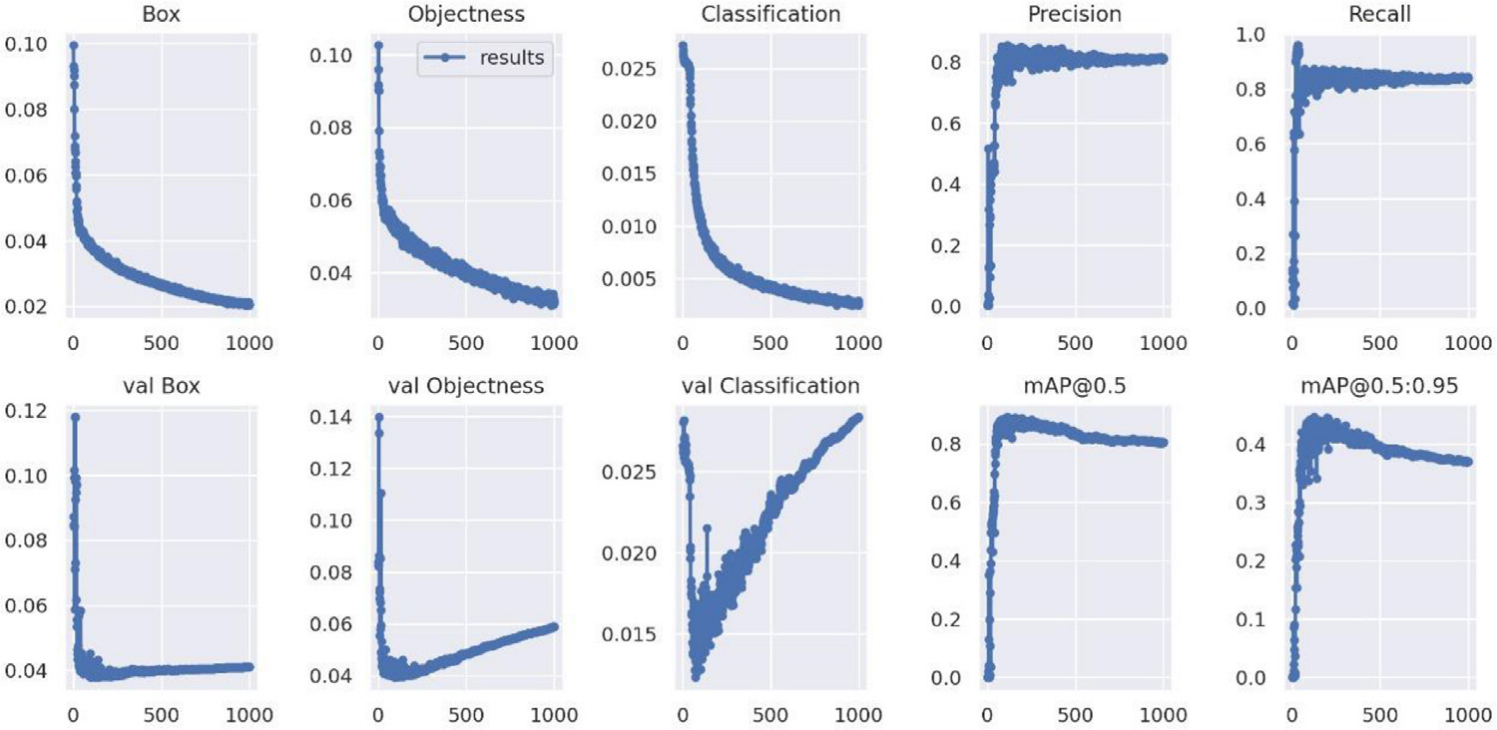
plots of box loss, objectness loss, classification loss, precision, recall and mean average precision (mAP) over 100 training epochs for the training and validation sets.

The bounding box for *Bactrocera Zonata* is represented by pink color with 86% confidence level and the bounding box for *Bactrocera Dorsalis* is represented by blue color with 91% and 87% confidence levels. The first three plots in Fig. 20 shows three types of loss: box loss, objectness loss, and classification loss. The box plot represents the performance of the model in locating the centre of an object and how well the predicted bounding box covers the entire object.

Objectness refers to the measure of probability that an object is present in the region of interest. High objectness means that the image window is likely to contain an object of interest. The third plot in top row shows the classification loss that gives an idea of how well the algorithm can predict the correct class of a given object. The first three plots in the bottom row shows validation box loss, validation objectness loss and validation classification loss respectively.

The model improved rapidly in terms of precision, recall and mean average precision that started declining after about 100 epochs. The box, objectness and classification losses of the validation data also showed a rapid decline until around epoch 100. We achieved the optimal weights by stopping early.

We have selected YOLOv5 as one of the object detection techniques to evaluate the quality of images in the dataset that proved to be reasonably good to be used for fruitfly species classification. Based on the results, we therefore believe that our current approach is appropriate for species detection and classification based on this dataset, and it could be further improved by using advanced deep learning methods. However, the main focus of the paper is image dataset description and evaluation of dataset for fruitfly detection and species classification and our dataset is reasonably applicable to any method that can perform object detection and classification.

How to Download?

The fruit fly species dataset has been uploaded and published on public repository Mendeley. Any user who wants to use this dataset for non-commercial purposes may use it after citing this publication. The dataset is available at http://dx.doi.org/10.17632/hgz2n5jxhp.1.

### 2.5 Mobile Application Development

**Fig. 21:**
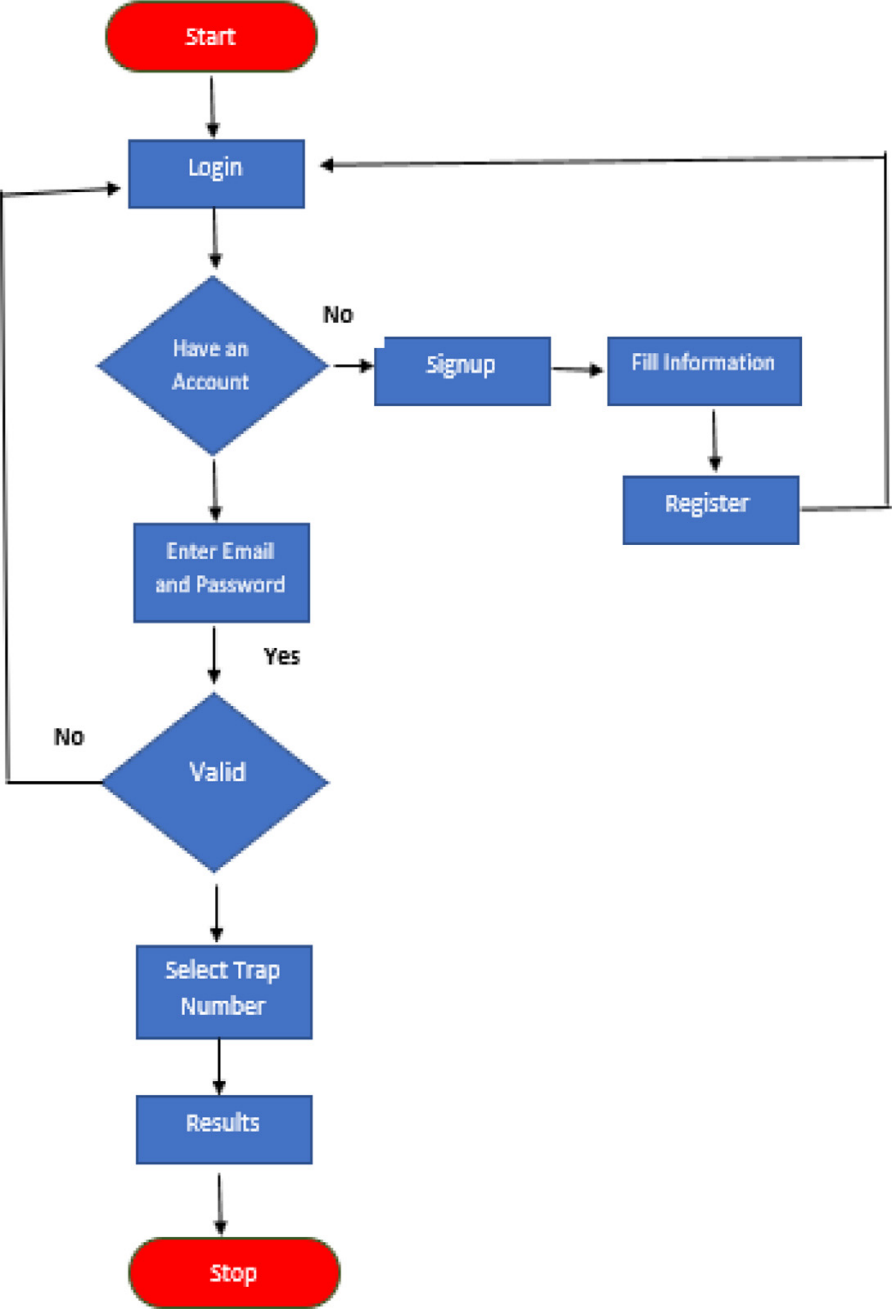
Mobile Application Workflow.

A mobile application has been developed to visualize the results of fruit fly species detection and recognition system that is directly connected to the data collected through the smart trap. This app is simple and easy to be used by farmer or a researcher. Local temperature and humidity values measured by IoT sensors are displayed along with images in real time. The workflow diagram of mobile application is shown in Fig. 21.

**Fig. 22:**
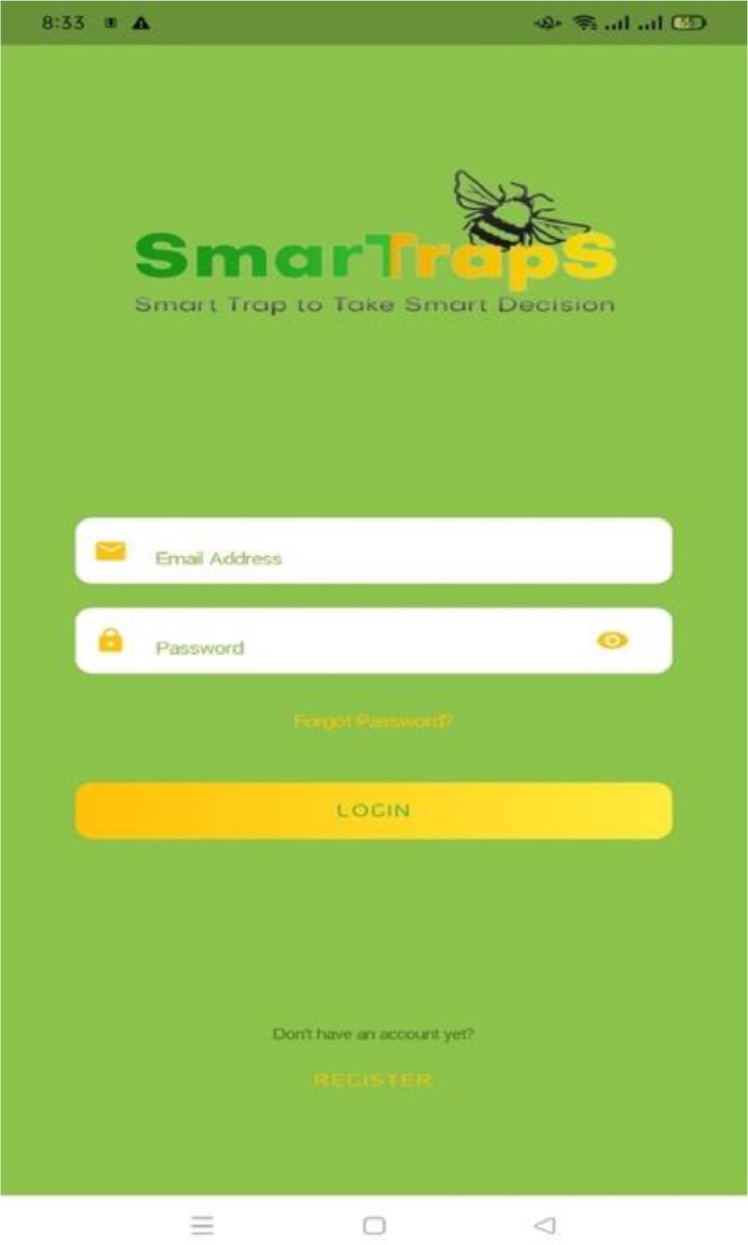
Login screen.

**Fig. 23:**
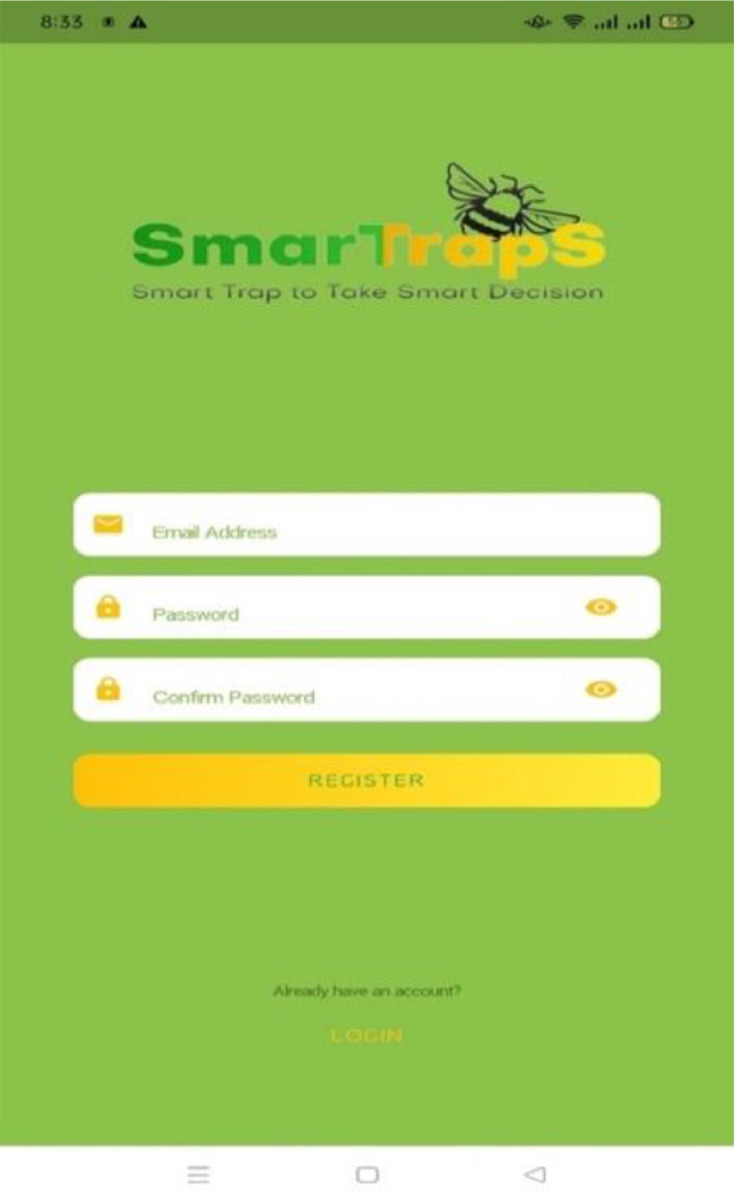
Registration details.

**Fig. 24:**
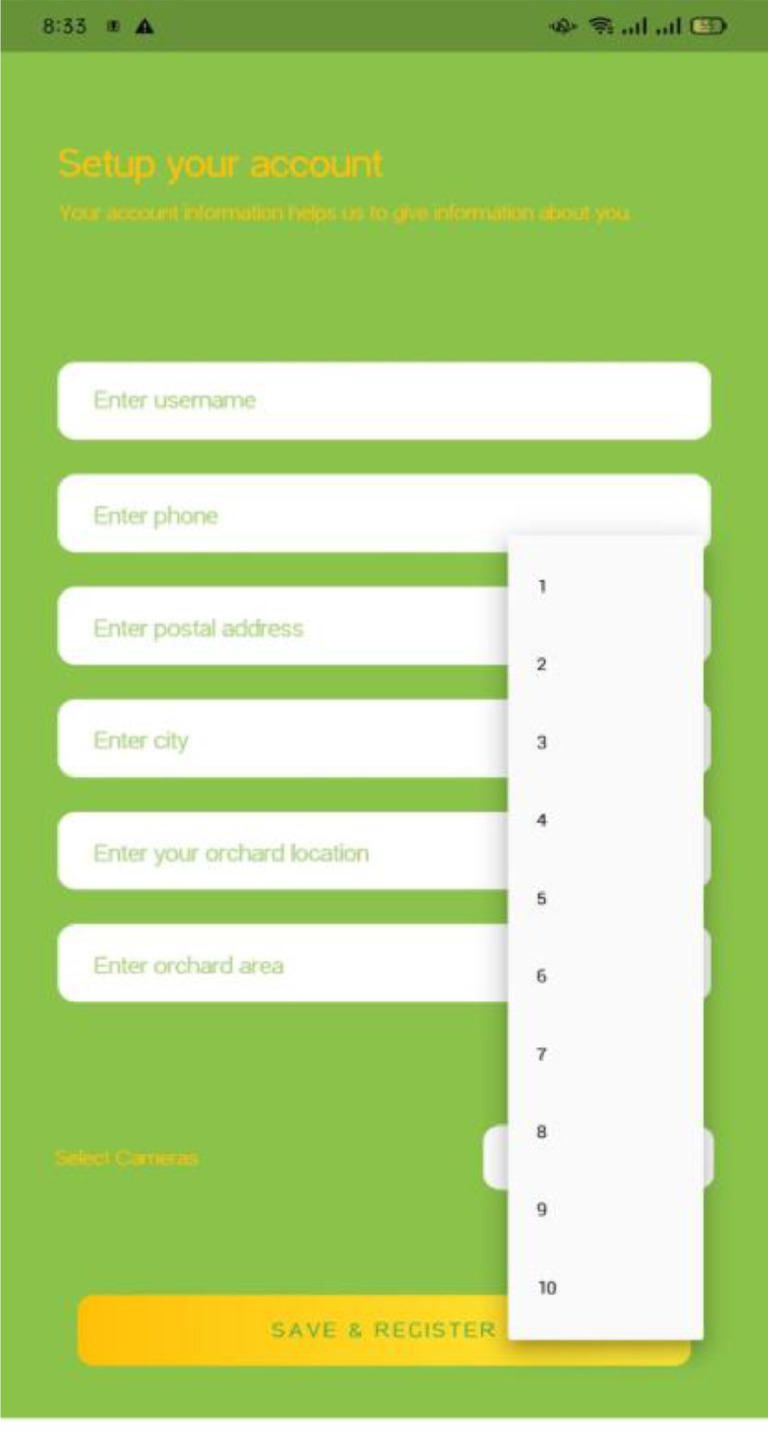
Signup screen.

**Fig. 25:**
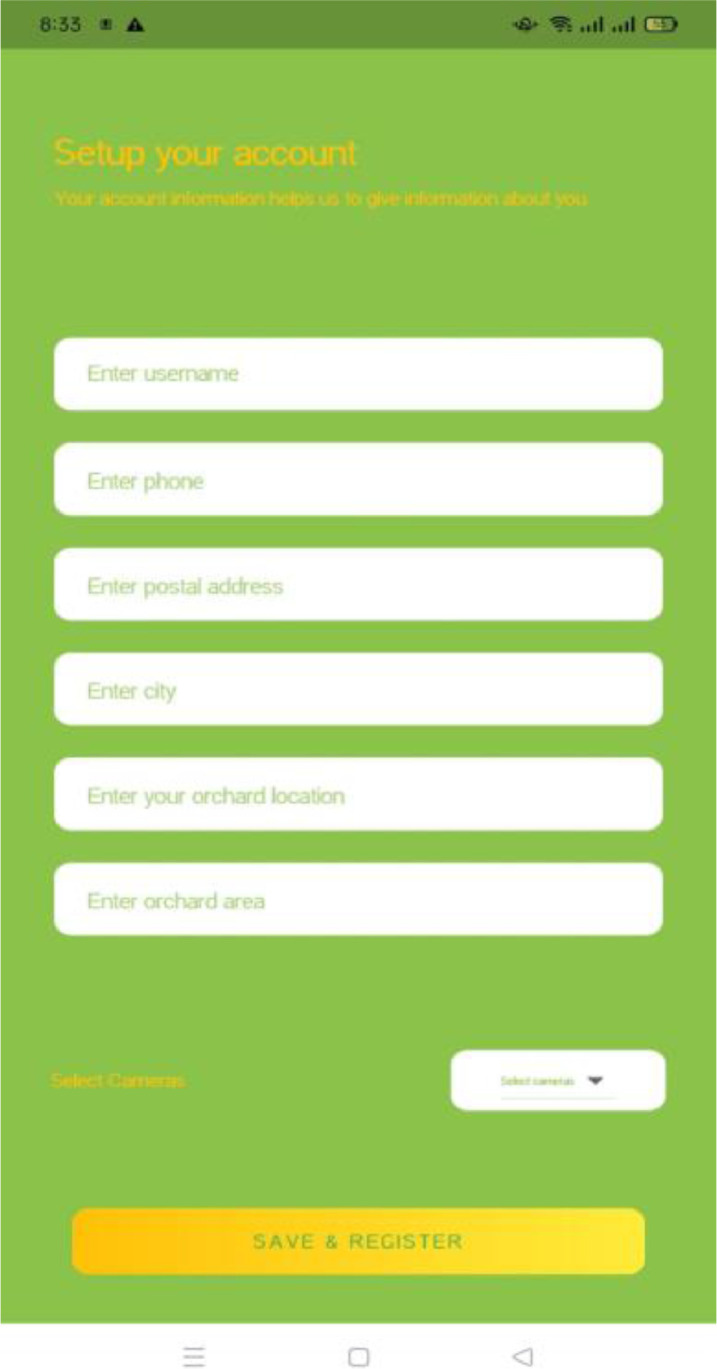
Camera selection.

Mobile application GUI:

Login and Signup screens of the mobile application is shown in Figs. 22 and 23. All users must be registered to use this application. The registration is done by providing contact details of farmer, address of orchard, area of orchard, and number of cameras to be used for fruitfly species monitoring (Figs. 24 and 25). After completing the registration, the user may login to the mobile application.

**Fig. 26:**
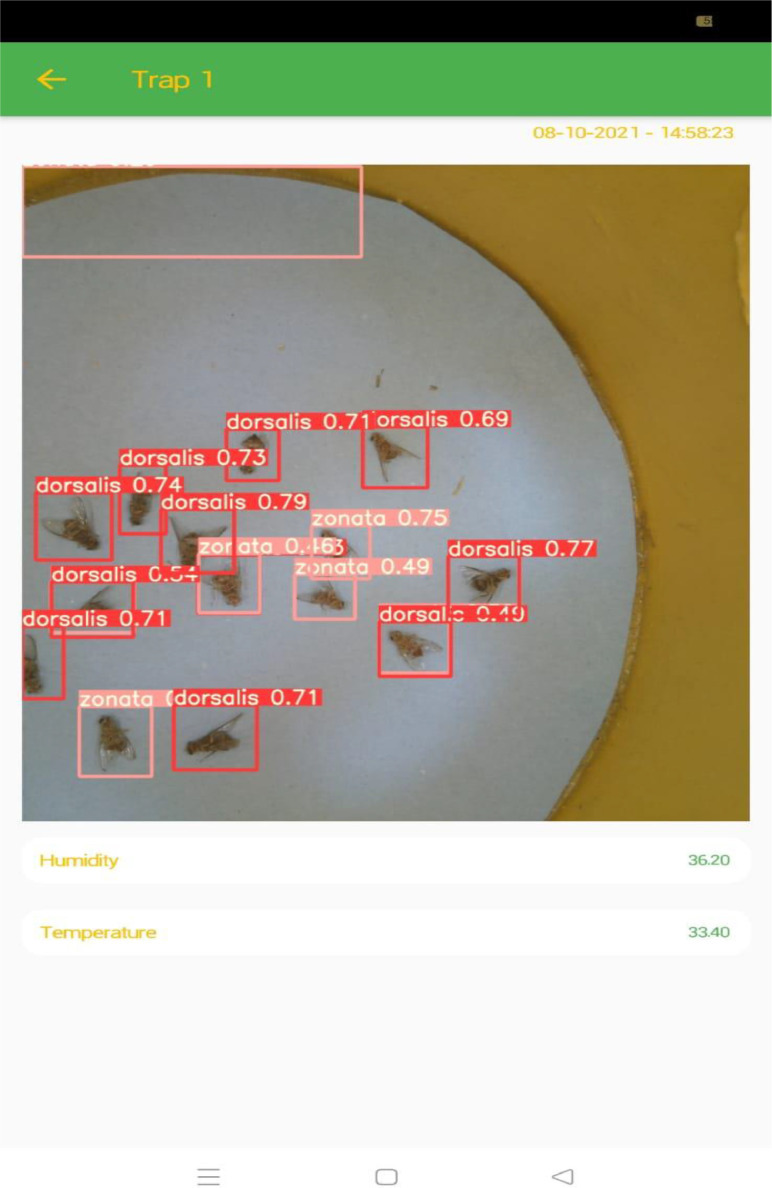
Results visualization through mobile application.

If the user selects one camera for monitoring, the application will provide the results of one camera only. The results are visualized as shown in Fig. 26, where the user can visualise the real time information about fruitfly species present in the orchard, temperature, and humidity level in the orchard using the mobile application.

## Credit Author Statement

**Sana Tariq:** Data curation, Writing, Mobile app development; **Ayesha Hakim:** Conceptualization, Methodology, Supervision; **Awais Ahmad Siddiqi:** IoT configuration, Technical investigation; **Muhammad Owais:** Database, Networking.

## Funding

This research did not receive any specific grant from funding agencies in the public, commercial, or not-for-profit sectors.

## Declaration of Competing Interest

The authors declare that they have no known competing financial interests or personal relationships that could have appeared to influence the work reported in this paper.

## Data Availability

An Image Dataset of Fruit fly Species (Original data) (Mendeley Data).
